# A Single-session Crisis Intervention Therapy Model for Emergency Psychiatry

**DOI:** 10.5811/cpcem.2018.10.40443

**Published:** 2019-01-10

**Authors:** Scott A. Simpson

**Affiliations:** Denver Health Medical Center, Psychiatric Emergency Services, Department of Behavioral Health, Denver, Colorado

## Abstract

Presentations for anxiety and depression constitute the fastest growing category of mental health diagnoses seen in emergency departments (EDs). Even non-psychiatric clinicians must be prepared to provide psychotherapeutic interventions for these patients, just as they might provide motivational interviewing for a patient with substance use disorders. This case report of an 18-year-old woman with suicidal ideation illustrates the practicality and utility of a brief, single-session, crisis intervention model that facilitated discharge from the ED. This report will help practitioners to apply this model in their own practice and identify patients who may require psychiatric hospitalization.

## INTRODUCTION

Symptoms of anxiety and depression are the most common reasons to present for emergency psychiatric care.^1^ The broad differential for depressive and anxiety symptoms includes major depressive, post-traumatic stress, adjustment, substance-induced, and personality disorders.^2^ Because medications are not indicated for some of these conditions, all emergency department (ED) clinicians must be prepared to provide brief, non-pharmacologic treatment. This case report demonstrates a single-session, crisis intervention model for ED patients presenting with anxiety and depression.

## CASE REPORT

An 18-year-old woman was brought to the ED by ambulance. Paramedics reported that the patient was on the phone with her mother and said she wanted to be dead. Her mother lives in another country and called emergency services. The patient was tearful and “very stressed” on arrival. Vital signs, a routine urine toxicology screen, and pregnancy test were unremarkable. She reported suicidal thoughts for about a week attributed to poor grades in college, family conflict, and financial obligations. She had missed several appointments with her therapist and prescriber and had recently run out of sertraline (Zoloft). She declined to provide her mother’s phone number.

The patient described a history of abuse at a young age. She had one prior psychiatric hospitalization after walking into traffic in a suicide attempt at age 15. Other episodes of self-harm started at age 10 and were non-suicidal in nature. Her biological father had minimal contact with the patient. Her grandmother had been diagnosed with schizophrenia. The patient denied access to firearms.

**Figure 1:**
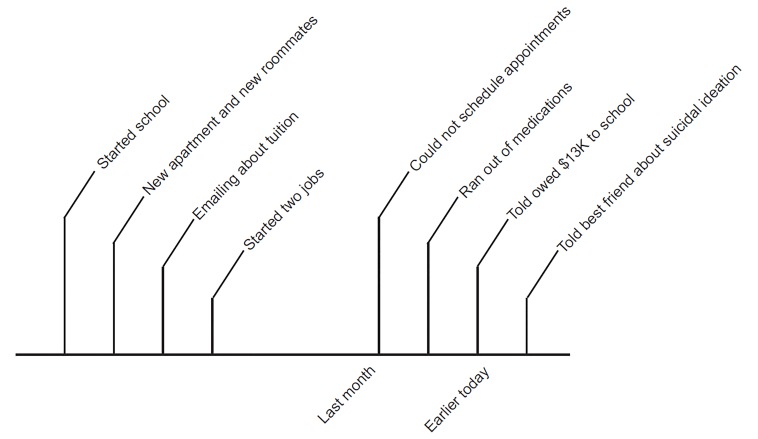
A timeline of stressors preceding the patient’s emergency department visit.

Concerned about multiple suicide safety risk factors, the emergency psychiatrist began a structured, single-session psychotherapy. The psychiatrist and patient wrote a timeline of events preceding the presentation (Figure 1). In so doing, she provided more details of her history. Ten months prior, she had to leave her apartment due to conflicts with roommates. Beginning college, she worried about tuition and found two jobs. Despite several attempts to re-schedule her therapy appointments around her work schedule, the therapist’s office did not return her calls. The patient also revealed that a supportive stepfather lived nearby. The morning of her ED visit, she received another reminder about her tuition bill. She was talking with a roommate about this bill; however, she felt her roommate did not fully appreciate her challenges, and she then called her mother.

The psychiatrist and patient agreed all this would be stressful for anyone. Her affect evolved from tearful to more composed, and she identified some immediate goals: find a new therapist; talk with her school about a tuition grant; identify a tutor; and spend more time doing things she enjoys (Supplemental Figure). She agreed to let the resident call her mother who could help complete these tasks. The patient’s mother corroborated the patient’s history. In fact, the mother had already spoken with the school about help with tuition and had begun searching for new outpatient providers.

To complete discharge planning, a nurse made an appointment for the patient with a new outpatient provider. The patient completed a written safety plan and was offered a follow-up call. The family was apprised of local crisis resources. Within an hour, the evaluating psychiatrist felt that this patient’s acute risk was significantly mitigated through safety planning, mobilization of social supports, connection to treatment, and acute de-escalation to justify discharge. After six months, she had persistent resolution of suicidal thoughts without recurrent self-harm or inpatient hospitalization.

## DISCUSSION

This brief psychotherapy emphasizes active problem-solving and is adapted from a multi-session model built for integrated care settings.^3^ Specialized single-session psychotherapies have been described for other psychiatric conditions including insomnia,^4^ gambling,^5^ agitation,^6^ and suicidal ideation.^7^ Therapy models described for ED settings are often applied by non-psychiatric staff, for example, motivational interviewing for substance use^8^ and safety planning for suicidality.^9^,^10^

This model uses the concept of crisis as a framework for assessment and treatment. A crisis occurs when a person’s usual coping skills are inadequate to a life stressor.^11^ A crisis may be precipitated by medical illness or interpersonal conflicts. A patient’s ability to cope with stressors arises from individual temperament, life experiences, personal skills, and social network. When a crisis develops, individuals are unable to access these strengths to resolve the crisis. Anxiety, depression, a sense of feeling overwhelmed, or suicidal ideation ensues in a patient with perhaps little psychiatric treatment history and a high level of functioning that includes stable employment and relationships. Some patients manifest primitive coping skills such as somatization that precipitate an ED visit. A crisis may also relate to worsening symptoms in patients with chronic psychiatric illness, for example, increased suicidal ideation in a patient with borderline personality disorder. Crisis does not fit neatly into the *Diagnostic and Statistical Manual of Mental Disorders*, 5^th^ edition, (DSM-5) but is most closely related to the diagnosis of adjustment disorder.^12^

Single-session therapy leverages the crisis model to help patients and providers understand the origins of the ED visit and begin actively resolving the crisis. This intervention may be delivered by emergency physicians or ED behavioral health consultants including social workers or nurses. Patients most likely to benefit from this therapy present in the context of a discrete life stressor and have a history of better psychological functioning and insight.

**CPC-EM Capsule**

What do we already know about this clinical entity?

*Anxiety, depression, and adjustment reactions represent the fastest-growing category of reasons for psychiatric presentations to the emergency department (ED)*.

What makes this presentation of disease reportable?

*This case demonstrates how a single-session crisis therapy in the ED may avert hospitalization for a depressed, suicidal patient*.

What is the major learning point?

*ED clinicians should be prepared to recommend or deliver brief psychotherapy for these common psychiatric presentations*.

How might this improve emergency medicine practice?

*Most suicidal ED patients are hospitalized. However, most patients with depression or suicidality can be safely treated in the ED and discharged*.

### One-Session Crisis Intervention Psychotherapy

The goals of this intervention include ameliorating anxiety and depressive symptoms, initiating treatment, and identifying patients who may need referral for more intensive psychiatric treatment. These steps and their therapeutic benefits are summarized in Table.

#### 1. Recognize the Crisis and Identify the Precipitant

Patients in crisis present to the ED with a range of psychiatric symptoms including anxiety, depression, fatigue, or poor sleep. After excluding a somatic etiology of psychiatric symptoms and ensuring acute safety, the clinician must elucidate the onset of the patient’s psychiatric symptoms. In the ED it is important to keep in mind that suicidality is often a symptom of underlying distress, does not necessarily indicate the presence of a severe psychiatric disorder, and can be treated in outpatient settings.^13^

Writing a timeline with the patient helps identify life stressors driving the crisis. This technique is helpful for several reasons. First, many patients in crisis feel overwhelmed and are challenged to recall and reconstruct a helpful history. A structured framework focuses the interview on the acute presentation. A timeline is easy for both clinicians and patients to interpret. And, in the act of writing a timeline together, the clinician and patient build therapeutic rapport that itself is part of the healing process. Finally, the resulting product can be used to later formulate the crisis state with the patient.

#### 2. Characterize the Patient’s Response

The patient’s emotional and behavioral responses to the crisis state should be considered in guiding treatment.^14^

Validate the patient’s emotional response to the crisis. The emotional response is often readily described by the patient: stressed, overwhelmed, anxious, or alone. The clinician may validate the emotional state by noting it to be an understandable response to the clear stressors described in the timeline. A patient’s endorsement of depression is not synonymous with major depressive disorder (a specific diagnosis with precise diagnostic criteria).

Behavioral responses are characterized by immobility, avoidance, or adaptation. Immobility is a sense of feeling stuck and persistently unable to problem-solve, as this patient felt initially. Some patients avoid their problems entirely, thereby prolonging the crisis and exacerbating its consequences. Immobile and avoidant patients need help identifying the precipitant of the crisis and brainstorming possible solutions. Immobile or avoidant patients who cannot demonstrate more adaptive skills may require referral to specialty psychiatric care.

Patients who demonstrate adaptive responses to crisis are positioned to grow from their crisis and manage their lives more effectively. In this case, the patient moved from a more immobile stance to one characterized by greater initiative and adaptation.

#### 3. Formulate Together

With a timeline of precipitants and a sense of the patient’s response styles, the clinician formulates the acute crisis aloud with the patient. What are the precipitants? How do these make the patient feel? What does the patient need to address the crisis? What choices are available?

The resulting conversation is both diagnostic and therapeutic. This patient experienced relief from an expert’s explanation of why she did not feel well. The clinician validated the severity of the patient’s stressors while offering optimism and active problem-solving.

A broad psychiatric differential should always be considered. Cognitive impairment related to severe depression or disorganization due to psychosis may be recognized in the course of therapy. Such symptoms complicate less-restrictive outpatient treatment and may alter disposition planning. Antidepressant discontinuation syndrome was considered less likely here given the timing and quality of her depressive symptoms.

#### 4. Identify Behavioral Goals and Offer Concrete Support

The clinician helps the patient generate a to-do list of goals to resolve the crisis. This patient’s list is included as Supplemental Figure. Goals should be specific, realistic, and accomplishable in the near future.^15^ Patients with more aspirational goals (e.g., feel better) should identify intermediate steps that are specific and accomplishable. Solutions-focused thinking can be introduced by asking, “If things were going well in your life, how would things look four weeks from now?” This conversation invites the patient to anticipate potential obstacles to resolution of the crisis—and also begin envisioning discharge from the ED.

Clinicians need to provide practical support for patients. For example, this patient needed help making an international phone call. Making an appointment for an outpatient provider improves outpatient adherence and reduces ED return rates.^16^,^17^ Identifying triggers for suicidal thoughts, coping skills, and supportive contacts through a safety plan reduces the risk of subsequent self-harm and improves symptom burden.^9^,^18^,^19^

#### 5. Engage Social Supports

Patients in crisis are quick to say they have nobody to help them when, in fact, supportive friends or family are indeed available. This social network should be mobilized while the patient is in the ED.

**Figure 2:**
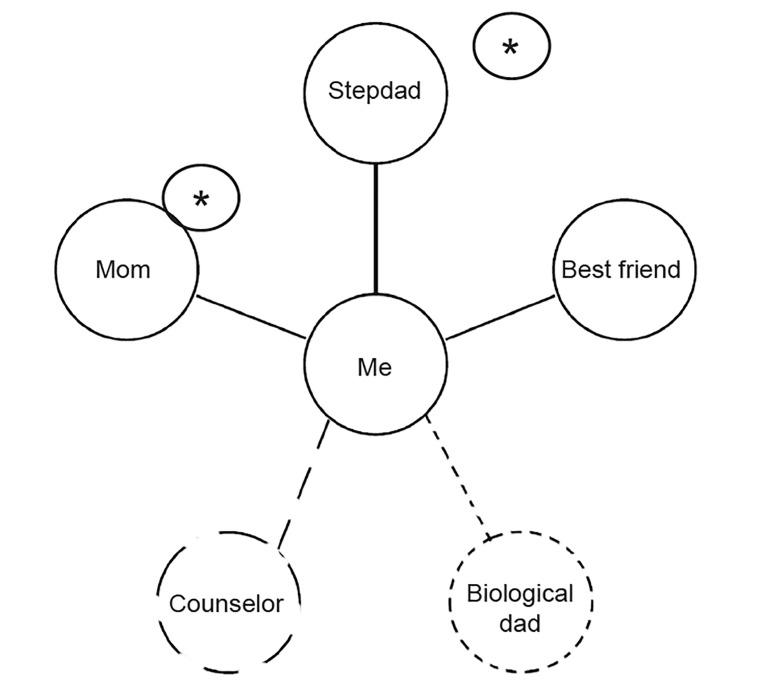
A hub-and-spoke diagram of the patient’s social supports.

A hub-and-spoke diagram helps the patient recognize persons who can help resolve the crisis (Figure 2). The patient is in the middle hub. As many other persons as possible are written around the spokes of the wheel. Supportive persons are connected to the hub with a solid line, and less-supportive contacts are connected with a dashed line. The most important one or two persons are starred.

The clinician should contact these supportive persons. Collateral information provides a stronger diagnostic and suicide safety assessment.^20^ In this instance, that the patient’s mother described so many supportive actions already underway illustrates how the crisis state induces a perception of isolation and hopelessness. Social supports should be enlisted to help in treatment planning. For example, family may take the patient to a follow-up appointment. When collateral information introduces new data worrying for safety risk or a social network is truly unavailable, the clinician may more strongly consider more intensive treatment including hospitalization.

## CONCLUSION

Most ED visits for suicidal ideation still result in hospitalization.^21^ This single-session, crisis intervention complements the traditional expectations of emergency psychiatric evaluations by providing clinicians a way to treat symptoms of anxiety and depression in the ED. This model may also assist in the treatment of boarding psychiatric patients and encourage further studies of psychotherapy in the emergency setting.

## Supplementary Information



## Figures and Tables

**Table:** Summary of working steps and therapeutic processes for one-session crisis intervention therapy.

Stage	Working steps	Therapeutic process
1. Recognize the crisis and identify the precipitant(s)	Assemble history Write timeline (Figure 1)	Ascertain more replete history Inform DSM-5 diagnosis Demonstrate active exploration of crisis Build rapport
2. Characterize the patient’s response	Describe emotional response Describe behavioral response: immobility, avoidance, or adaptation Immobile patients benefit from naming the precipitant; avoidant patients benefit from identifying solutions	Validate emotional state Validate therapeutic relationship Consider whether lack of adaptive posture requires higher level treatment Support evolution towards adaptive response in course of session
3. Formulate together	Discuss what is going on: What are the precipitants? How does the patient feel? What does the patient need? What choices are available? What’s going well despite the crisis? Agree on an explanation for symptoms and the ED visit	Validate the work done in prior steps Align with patient on identifying the problem Begin problem-solving
4. Identify behavioral goals and offer concrete support	Write a list of goals (Supplemental figure) For easy “to-do’s,” provide concrete support (e.g., make appointments, help with phone calls) For more aspirational goals (e.g., “feel better”), identify intermediate, actionable steps Safety plan Restrict access to lethal means Arrange follow-up call if possible	Demonstrate adaptive problem-solving Begin envisioning discharge and anticipating challenges Reduce safety risks
5. Engage social supports	Write hub-and-spoke diagram of social supports (Figure 2) Call social supports for collateral information Enlist supports in discharge planning	Enhance social connectedness Improve concrete support for discharge plan Increase likelihood of other persons referring patient to treatment should crisis worsen

*DSM-5*, Diagnostic and Statistical Manual for Mental Disorders, 5th ed.; *ED*, emergency department.
