# Supplementary figures and images for: A Single-session Crisis Intervention Therapy Model for Emergency Psychiatry
Source: Clin Pract Cases Emerg Med. 2019 Jan 10;3(1):27–32. doi: 10.5811/cpcem.2018.10.40443 (PMC6366378; doi:10.5811/cpcem.2018.10.40443)

**Supplemental Figure.** A to-do list with goals written by the patient.


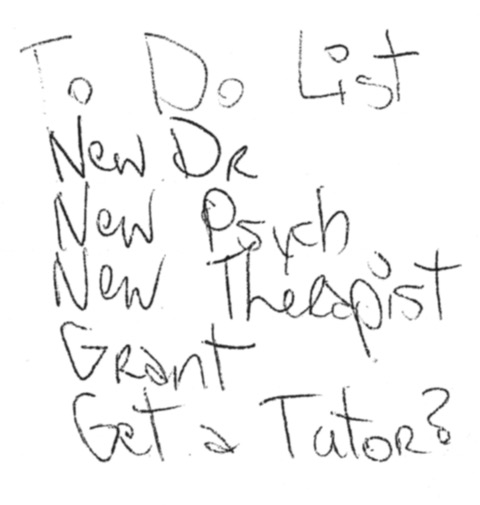

Supplement: Supplementary file 1 [file cpcem-03-27-s001.docx]
